# New Revelations Ignite the EXCEL Affair and Expose the Distortion of Science

**DOI:** 10.21470/1678-9741-1-2020-0609

**Published:** 2020

**Authors:** Walter J. Gomes, Luciano C. Albuquerque, Rui M.S. Almeida

**Affiliations:** 1 Cardiovascular Surgery Discipline and São Paulo Hospital. Escola Paulista de Medicina. Federal University of São Paulo, São Paulo, Brazil.; 2 São Lucas Hospital of the Pontifical Catholic University of Porto Alegre, Porto Alegre, RS, Brazil.; 3 Faculty of Medicine of the University Center Assis Gurgacz, Cascavel, PR, Brazil.

New printed evidence reignited the controversy over the EXCEL affair, exposing more important flaws, both in the trial’s statistical analysis and the revelation that the previously concealed myocardial infarction data has now been made available.

Further to the extensive disclosure of flagrant bias introduced into the design, analysis, and publication of the 5-year follow-up of the EXCEL trial^[1]^, the June edition of the Journal of American Medical Association (JAMA) Internal Medicine revealed and highlighted that the formal hypothesis testing, which in the EXCEL trial was prespecified as a demonstration of noninferiority at 3 years, was without discussion or explanation switched to a superiority test in the 5-year analysis^[2]^. If as prespecified, the same noninferiority analysis had been conducted at 5 years it would not have met the criteria for statistical significance, and the printed conclusion would not have been possible. This is a classic case of interpretation bias, otherwise known as spin.

The newly identified violation follows in the wake of the controversy over data manipulation in the EXCEL trial. The article authored by Professor James M. Brophy, who is a professor of Medicine, Epidemiology, and Biostatistics at McGill University in Montreal, Canada, re-examines the design and results of the EXCEL trial and other recent randomized clinical trials related to the left main coronary artery disease (LMCAD) using Bayesian methods^[2]^.

The article shows furtive aspects and details of the statistical analysis, not previously acknowledged, that in effect concealed and circumvented the real results of the EXCEL trial to attain the desired conclusion. The method of statistical analysis used to interpret the 5-year data was different from that used in the 3-year publication, and in direct contrast to the original protocol, without explanation or justification. If EXCEL’s 5-year results had been interpreted in alignment with its original primary non-inferiority design of a pre-specified 4.2% primary result margin, a different conclusion would have been reached, even within the context of a standard analysis. Instead, the EXCEL authors construed the 5-year results as a superiority analysis, in which the null hypothesis did not consist of any differences so that the observed data was not extreme enough to reject that null hypothesis. The Bayesian analysis of the EXCEL primary outcome estimated with 95% probability that the 5-year primary outcome difference was increased with PCI compared with CABG and 87% probability that this difference was greater than one extra event per 100 patients treated. Given that in EXCEL these were relatively young patients with a mean age of 66 years (and therefore a potential life expectancy of 15-20 years) and that the statistically significant observed increase in mortality of 38% was actively accelerating raises real clinical concern. For the secondary composite endpoint, which also included repeated revascularizations, there were estimated probabilities of 98% for at least 4 extra events and of 90% for at least 5 extra events per 100 patients treated with PCI. Therefore, PCI was associated with less favorable results for all events, including mortality, compared to CABG in patients with LMCAD.

Professor Brophy, although recognizing that these analysis failures by the authors of the EXCEL trial may have occurred due to carelessness and/or incompetence of the authors, questions if sponsorship by the company that produced the stent used in the study led to more favorable results and conclusions. He reinforces that fourteen of 34 EXCEL trial authors, including the first and last authors, had a relationship with the study sponsor (the stent maker), and that 8 authors listed an affiliation with the Cardiovascular Research Foundation (CRF) which oversaw the study. CRF received a donation of US$ 937,000 from the sponsor during the study and Professor Brophy infers that these factors may have contributed to the entirely speculative hypothesis that these conflicts of interests contributed to the biased interpretation of EXCEL^[2]^.

In the same issue of JAMA Internal Medicine, a commentary by Professor Sanjay Kaul (Professor of medicine at Cedars-Sinai Medical Center in Los Angeles) raises the same concerns about the inappropriateness of changing the protocol specified statistical analysis from non-inferiority at 3 years to superiority at 5 years^[3]^. The reason for this change is not justified or even explained in the final EXCEL trial manuscript. However, it emphasizes that, if the authors had maintained the non-inferiority analysis that was performed in 3 years (with a pre-specified upper margin of 4.2%), it would not meet those existing criteria at 5 years because the upper limit of the 95% difference at 6.5%, now exceeds that non-inferiority margin. He reiterates that the conclusion of EXCEL that PCI and CABG were similar, despite a 3.1% difference in mortality that favors CABG, is a classic example of interpretation bias, also known as spin. Spin is recognized to occur frequently when associated with commercial study sponsorship^[3,4]^.

The disclosure of alteration in the statistical methodology in the 5-year EXCEL analysis, making possible the printed conclusion, is very worrisome in view of its potentially adverse implications for patient safety. Likewise, the earlier claim from the EXCEL trial authors that the per-protocol Universal Definition of Myocardial Infarction data analysis, which was prespecified as a secondary endpoint, had been deemed ‘impossible’ because troponin was not properly collected, was overly refuted by the unveiling of the definition of the Third Universal Definition of Myocardial Infarction, which clearly states that if a cTn assay is not available, the best alternative for analysis is CKMB (measured by mass assay)^[1,5]^.

Despite the prior assertions of the EXCEL authors that no such data was available, in a new publication from the EXCEL trial authors, compulsorily replying to letters to New England Journal of Medicine (NEJM), now reported the protocol specified analysis of Third Universal Definition of Myocardial Infarction, based on CKMB data. This revealed a stark difference from the original paper, by now demonstrating the higher rate of periprocedural myocardial infarction related to PCI. And therefore, nullifying the original conclusion published in the NEJM^[6]^.

The authors claim that the difference in all-cause death was driven by an increase in non-cardiovascular death, due to late malignancies and sepsis, and in the absence of any obvious biologic plausibility is therefore likely due to chance. However, this denotes unfamiliarity with the basic principles of pathophysiology of coronary artery disease and the repercussion of distinctive revascularization therapies^[7]^. Mounting evidence implicates inflammation as a key player in many, if not all, diseases of the human body. The presence of a metallic intracoronary stent, acting as a foreign body inducing formation of a cardiac granuloma, elicits a local and systemic chronic inflammatory reaction, leading to a perennial low-grade inflammation with persistently higher blood TNF-alpha and interleukin-6 expression^[8-10]^. Inflammation has long been recognized as a key component of carcinogenesis and chronic low-grade inflammation, characterized by elevated concentrations of IL-6, TNF-α, and C-reactive protein, increase susceptibility to cancers and infections^[11-14]^. Additionally, drug-eluting stents actively releasing antiproliferative drugs promote chronic inflammation and determine a greater endothelial dysfunction and neoatherosclerosis^[15]^, which is associated with an long-term increased risk of solid-tumor cancer^[16]^.

As external and independent denouncements of the conduct and interpretation of the EXCEL trial are escalating, why do the EXCEL authors, of whom many are distinguished doctors, still remain silent over these crucial facts and the implication of potential real harm to patients? And what about the implications for guidelines that may have been or could in the future be based on such a flawed trial and conclusion?

Furthermore, the EXCEL trial has generated dozens of offspring manuscripts. Can we now trust these manuscripts if the primary manuscript was so severely flawed? And what about the patients enrolled in the EXCEL trial, who committed their trust and safety, to participate in a trial professedly created to help save lives? And how they might be now frustrated and even feeling betrayed by outcomes skewed for not confessed reasons?

Until these issues are addressed the EXCEL trial has the potential to become a deplorable example of distrust as a distorted form of science, leaving both the public and the medical profession to question how this happened and how to ensure that it cannot happen again.

While it would be hoped that the serious flaws of the EXCEL trial occurred through ignorance and/or incompetence it still raises the question of whether the EXCEL affair may have been driven by other conflicts both by the investigators and the company. The data generated by the EXCEL trial, the largest ever trial on this subject, is crucial for science and medical practice to understand the impact of the competing therapies of PCI and CABG in this deadly condition. Accordingly, and to restore trust both for patients and the medical profession the EXCEL data must be made open for unbiased and truthful thorough scrutiny, managed by independent reviewers. In the meantime, and given these very serious flaws the NEJM should issue a temporary retraction to prevent patients from being submitted to serious potential harm.
